# The Relationship Between C-Reactive Protein (CRP) Concentrations and Erythropoietin Resistance, Hospital Admission Rate, Control of Mineral Metabolism, and Comorbidity in Hemodialysis Patients

**DOI:** 10.7759/cureus.48793

**Published:** 2023-11-14

**Authors:** Reema Alsogair, Haifa A Altoub, Meznah Alsanad, Malak Alshukr, Bashayer AlObaid, Abdulla Al Sayyari, Mubarak Abdalla

**Affiliations:** 1 Medicine and Surgery, College of Medicine, King Saud bin Abdulaziz University for Health Sciences, Riyadh, SAU; 2 College of Medicine, King Saud bin Abdulaziz University for Health Sciences, Riyadh, SAU; 3 Rheumatology, King Saud bin Abdulaziz University for Health Sciences, Riyadh, SAU; 4 Nephrology, King Abdulaziz Medical City, Riyadh, SAU

**Keywords:** hd (hemodialysis), mineral metabolism, erythropoietin, chronic kidney disease (ckd), crp: c-reactive protein

## Abstract

Background

End-stage kidney disease patients undergoing hemodialysis are prone to develop inflammation detected by high serum C-reactive protein (CRP) levels. This study highlights the association between CRP and the erythropoietin resistance index, hospital admission rate, control of mineral metabolism, and comorbidities in a tertiary hospital and two dialysis centers in Saudi Arabia.

Objectives

The objective of the study is to assess the relationship between CRP levels and hemoglobin level, hospital admission rate, mineral metabolism, and comorbidity in hemodialysis patients.

Materials and methods

This was a cross-sectional study conducted at King Abdulaziz Medical City Dialysis Center and the South and North Dialysis Centers of King Abdullah Dialysis Program in Riyadh. All hemodialysis adult patients who have been on dialysis for over six months were included. Patients with acute illnesses and pediatric patients were excluded. The association between CRP and other variables was reported using the Pearson correlation test. The calculated sample size was 218 by using the Raosoft website; however, the final number we analyzed was 209 after exclusion.

Results

The prevalence of a high level of CRP was more common among patients with diabetes mellitus (p=0.008) and those who were using antihypertensives (p=0.044) while the prevalence of a high level of CRP was less common among underweight patients (p=0.031) and hepatitis C virus (HCV)-positive patients (p=<0.001). The mean value of Kt/V was significantly lower among patients with a high level of CRP (p=0.009). HCV negative was the only independent significant risk factor associated with high CRP concentration (p=0.006).

Conclusions

In conclusion, there was an association between CRP levels with BMI, diabetics, the use of antihypertensive medications, and negative or undetectable HCV test results with the latter being the only independent significant factor. These data suggest that patients meeting these characteristics are in an inflammatory state and more prone to develop complications; thus, implementing CPR testing in this population might be useful. Other reviews showing causations are needed to further elucidate.

## Introduction

Chronic kidney disease (CKD), as described in the Kidney Disease Improving Global Outcomes (i.e., “KDIGO”) guidelines, is either abnormal kidney function or structure that persists for more than three months and is confirmed by a glomerular filtration rate (GFR) < 60 mL/min/1.73 m^2^ or by markers of kidney damage, such as urine sediment(s) and electrolyte irregularities [1]. Based on a systematic analysis of studies published between 1990 and 2017, the number of cases of CKD recorded globally in 2017 was 697.5 million, with an increase of 29.3% from 1990 [2]. When the GFR drops to < 15 mL/min/1.73 m^2^, hemodialysis (HD) is indicated and is diagnosed as end-stage kidney disease (ESKD) [1]. Locally, the Saudi Centre for Organ Transplantation has estimated the number of patients undergoing HD to be 19,522. The high incidence of ESKD contributes to a significant disease burden [3]. Patients undergoing HD are prone to developing inflammation that can be detected using a highly sensitive C-reactive protein (CRP) test, which detects high levels of plasma CRP [1,4,5]. A CRP level ≥ 200 μg/ml is indicative of poor prognosis based on specific factors [1,6], which include erythropoietin resistance, hospital admission rate, uncontrolled mineral metabolism, and comorbidities [6,7]. High serum CRP levels have been identified as a predictor of decreased responsiveness to erythropoietin therapy in HD patients presenting with anemia [7,8]. Another factor associated with high CRP levels is uncontrolled mineral metabolism [6], which manifests as decreased levels of vitamin D (25(OH)D), which results in increased bone turnover and decreased calcium absorption [8,9].

Extraskeletal calcification is another manifestation of uncontrolled mineral metabolism that is also predicted by elevated CRP level(s) [6,10,11]. A recent study from Saudi Arabia reported that the prevalence of vascular calcification in patients undergoing HD was as high as 50% [10]. This, in turn, results in a higher risk of developing cardiovascular comorbidities, as established by several studies [12].

There have been no adequate studies investigating the relationship between hospital admission rate and CRP levels in patients undergoing HD. These factors result in a poor prognosis for HD patients and predict the severity of disease progression. However, the relationship between these factors and CRP levels is not fully understood and requires further characterization. As such, the present study examined the association between these factors. Moreover, our investigation assessed whether CRP could be a routine test based on findings and aimed to evaluate the relationship between the CRP level and erythropoietin resistance index (ERI), hospital admission rate, control of mineral metabolism, and comorbidities in patients undergoing HD at King Abdulaziz Medical City Dialysis Center and South and North Dialysis Centers of King Abdullah Dialysis Program in Riyadh, Saudi Arabia.

## Materials and methods

Study design and settings

The present investigation was designed as a quantitative cross-sectional study and was conducted from January to March 2020 at the King Abdulaziz Medical City Dialysis Center and the South and North Dialysis Centers of the King Abdullah Dialysis Program, Riyadh, Saudi Arabia. These public dialysis centers serve patients at The National Guard Hospital. Eligible patients included Saudi Arabian National Guard (SANG) military personnel, civilians, and their eligible dependents, non-Saudi employees working with SANG and their eligible dependents, and King Saud bin Abdulaziz University for Health Sciences Students and interns, their spouses, and children.

Study participants

IRB approval was obtained from the IRB office at King Abdullah International Research Center prior to the data collection process. A total of 209 patients (median age, 50 years) were included, 52% of whom were male and 48% were female. All stable adult patients at King Abdulaziz Medical City and the South and North Dialysis Centers of King Abdullah Dialysis Program, who had been undergoing HD for > 6 months were included, whereas patients with an acute illness were excluded because acute illnesses cause an increase in CRP level(s) [13]. All 209 subjects were selected using a non-randomized consecutive sampling technique for the descriptive purposes of the study.

Data collection process

Data were collected using chart review, and patient data were reviewed and extracted from the BESTCare system in electronic form and entered into a spreadsheet file (Excel, Microsoft Inc, Redmond, WA, USA). CRP was the independent variable, whereas body mass index (BMI), dialysis vintage, calcium and phosphate levels, ERI, hospital admission rate, and Charlson Comorbidity Index (CCI) were all dependent factors. The CCI categorizes patient comorbidities based on the International Classification of Diseases (ICD). The ERI was calculated by dividing the weekly body-weight-adjusted erythropoietin dose by hemoglobin concentration and determined monthly to assess resistance to erythropoietin treatment. CRP levels > 200 μg/ml were considered to be high. In addition, the diagnosis of diabetes mellitus (DM) and hypertension and the calculation of Kt/V were determined by reviewing patient medical files using the BestCare system; all other variables were collected in the same manner.

Statistical analysis 

Data were analyzed using IBM SPSS Statistics for Windows, Version 26 (Released 2019; IBM Corp., Armonk, New York, United States). Continuous variables are expressed as mean and standard deviation. Descriptive statistics are expressed as number, percentage, mean, and standard deviation, as appropriate. The association between CRP levels and the ERI, hospital admission rate, control of mineral metabolism, and comorbidities was determined using the Pearson correlation test. Analyses examined the relationship between CRP level at baseline and clinical characteristics of patients undergoing HD using the chi-squared test (categorical variables) and independent sample t-test (continuous variables). Multivariate regression analysis was performed to determine independent significant predictor(s) of high CRP levels and expressed as OR with corresponding 95% CI. Differences with p < 0.05 were considered to be statistically significant.

## Results

Data from 209 patients undergoing HD were used to determine the relationship between high CRP levels and erythropoietin resistance, hospital admission rate, control of mineral metabolism, and comorbidities. As reported in Table 1, the most common age group was > 70 years (40.2%), and more than one-half were female (51.7%). With regard to BMI, more than one-third had normal BMI (35.9%) others were overweight (28.2%), and obese (28.2%). DM was the most common cause of CKD (75.6%). The proportion of patients undergoing antihypertensive drug therapy was 71.8%, with a frequency of use of 1 to 2 times per day (90.7%). Similarly, history of stroke, myocardial infarction (MI), cardiovascular disease, and heart failure were present in 25.4%, 19.6%, 45.5%, and 38.3% of the cohort, respectively. Similarly, 6.7% of patients were hepatitis C virus (HCV)-positive, and 6.7% were hepatitis B virus HCV-positive. The most common dialysis method was HD (76.2%), and the most common type of vascular access was a permanent catheter (PC) (58%) (Table 1).

**Table 1:** Baseline characteristics of the patients at King Abdulaziz Medical City and the South and North Dialysis Centers of King Abdullah Dialysis Program (n=209) CKD: Chronic kidney disease; DM: diabetes mellitus; MI: myocardial infarction; HDF: hemodiafiltration; HD: hemodialysis; AVG: arteriovenous graft; PC: permanent catheter

Study Variables	N (%)
Age group	
≤50 years	45 (21.5%)
51 – 70 years	80 (38.3%)
>70 years	84 (40.2%)
Gender	
Male	101 (48.3%)
Female	108 (51.7%)
BMI level	
Underweight (<18.5 kg/m^2^)	16 (07.7%)
Normal (18.5 – 24.9 kg/m^2^)	75 (35.9%)
Overweight (25 – 29.9 kg/m^2^)	59 (28.2%)
Obese (≥30 kg/m^2^)	59 (28.2%)
Causes of CKD	
Not DM	51 (24.4%)
DM	158 (75.6%)
On antihypertensive therapy	
No	59 (28.2%)
Yes	150 (71.8%)
Number of times taking antihypertensive therapy ^(n=150)^	
1 – 2 times	136 (90.7%)
>2 times	14 (09.3%)
History of stroke	
No	156 (74.6%)
Yes	53 (25.4%)
History of MI	
No	168 (80.4%)
Yes	41 (19.6%)
History of cardiovascular disease	
No	114 (54.5%)
Yes	95 (45.5%)
History of heart failure	
No	129 (61.7%)
Yes	80 (38.3%)
HCV positive	
No	195 (93.3%)
Yes	14 (06.7%)
HBV positive	
No	195 (93.3%)
Yes	14 (06.7%)
Type of dialysis therapy	
HD	157 (76.2%)
HDF	49 (23.8%)
Type of vascular access	
AVF	83 (40.1%)
AVG	04 (01.9%)
PC	120 (58.0%)

Furthermore, the most frequent measure of ejection fraction was 46-55% (68.3%). Incidentally, the prevalence of diastolic dysfunction was 49.3%, while the prevalence of normal right and left atrial function was 78% and 38.8%, respectively. Moreover, the proportions of patients with hypokinesia and pseudo-normalization were 25.4% and 18.7%, respectively. Conversely, 59.8% had been hospitalized in the past 12 months, with infection being the most common cause (33.5%), and 30.1% reported having visited > 1 hospital. In addition, 80.8% of the patients were diagnosed with high CRP level(s) (Table 1).

Differences in clinical and laboratory characteristics in relation to CRP levels are summarized in Table 1. The mean pre-dialysis systolic and diastolic blood pressure for the last three sessions were 145 mmHg and 66.9 mmHg, respectively, while in post-dialysis, the respective values for the last three sessions were 137.5 mmHg and 65 mmHg. Mean weight gain for the last three dialysis sessions was 1.98 kg. The mean CCI was 6.70, while the mean value of the calculated ERI was 41.8 IU/kg/w/g/dL and the erythropoietin dose per week was 81.2 units/kg. The mean hemoglobin, calcium, phosphorous, sodium, serum albumin, total cholesterol, pre-dialysis serum creatinine, and parathyroid hormone (PTH) levels were 108.4 pg/ml, 2.23 pg/ml, 1.49 pg/ml, 133.8 pg/ml, 34.5 pg/ml, 3.71 pg/ml, 647.4 pg/ml, and 64.4 pg/ml, respectively. Additionally, the mean value of the Ca × P product was 3.32, whereas the mean value of the Kt/V was 1.22. When compared with the CRP level, the mean value of Kt/V was significantly lower in patients with a high CRP level (p=0.009) (Table 2). 

**Table 2:** Differences of clinical and laboratory characteristics in relation to the CRP level of the patients at King Abdulaziz Medical City and the South and North Dialysis Centers of King Abdullah Dialysis Program PTH: Parathyroid hormone; SBP: systolic blood pressure; DBP: diastolic blood pressure; CRP: C-reactive protein

Variables	Overall Mean ± SD	High Mean ± SD	Normal Mean ± SD	p-value
Mean predialysis SBP of the last three dialysis sessions (weekend sessions)	145.0 ± 22.1	145.0 ± 21.9	145.9 ± 24.3	0.818
Mean predialysis DBP of the last three dialysis sessions (weekend sessions)	66.9 ± 13.8	66.3 ± 13.6	69.3 ± 15.0	0.229
Mean postdialysis SBP of the last three dialysis sessions (weekend sessions)	137.5 ± 18.6	136.6 ± 19.3	139.5 ± 16.1	0.408
Mean postdialysis DBP of the last three dialysis sessions (weekend sessions)	65.3 ± 11.7	64.7 ± 11.4	68.2 ± 12.7	0.100
Mean weight gain in the last three dialysis session (weekend session)	1.98 ± 1.06	1.96 ± 1.14	2.13 ± 0.75	0.394
Charlson comorbidity index	6.70 ± 2.89	6.92 ± 2.86	5.75 ± 3.05	0.063
Calculated Erythropoietin Index	20.5 ± 30.4	18.4 ± 11.7	26.9 ± 51.4	0.070
Erythropoietin dose per week	41.8 ± 26.7	40.7 ± 26.5	40.6 ± 25.2	0.976
Hb level	108.4 ± 19.1	109.1 ± 18.9	107.7 ± 20.8	0.689
Ca level	2.23 ± 0.25	2.23 ± 0.26	2.22 ± 0.21	0.800
Phosphorus level	1.49 ± 0.54	1.50 ± 0.56	1.53 ± 0.49	0.791
Na level	133.8 ± 3.92	133.9 ± 3.88	133.3 ± 4.16	0.334
Serum albumin level	34.5 ± 4.46	36.6 ± 4.43	36.9 ± 4.55	0.745
Total serum cholesterol	3.71 ± 0.98	3.67 ± 0.99	3.88 ± 0.91	0.223
Predialysis serum creatinine	647.4 ± 256.4	656.8 ± 257.3	664.1 ± 242.2	0.874
PTH level	64.4 ± 55.7	65.9 ± 59.6	60.6 ± 40.4	0.607
Ca X P product	3.32 ± 1.33	3.34 ± 1.37	3.35 ± 1.17	0.957
Kt/V	1.22 ± 0.28	1.19 ± 0.28	1.32 ± 0.28	0.009 **

When measuring the relationship between the CRP level and baseline patient characteristics, a high CRP concentration was more common among patients with DM (p = 0.008) and those undergoing antihypertensive therapy (p = 0.044), while the prevalence of high CRP concentrations was less common among those who were underweight (p=0.031) and those who were HCV-positive. However, differences in age group (in years), sex, history of stroke, history of MI, history of cardiovascular disease, history of heart failure, hepatitis B virus positivity, type of dialysis therapy, and type of vascular access were not statistically significant (all p>0.05) (Table 3).

**Table 3:** Relationship between the level of CRP and the baseline characteristics of the patients at King Abdulaziz Medical City and the South and North Dialysis Centers of King Abdullah Dialysis Program (n=198) CKD: Chronic kidney disease; DM: diabetes mellitus; MI: myocardial infarction; HDF: hemodiafiltration; HD: hemodialysis; AVG: arteriovenous graft; PC: permanent catheter; CRP: C-reactive protein

Factor	High N (%) ^(n=160)^	Normal N (%) ^(n=38)^	p-value
Age group			
≤50 years	32 (20.0%)	12 (31.6%)	0.098
51 – 70 years	57 (35.6%)	16 (42.1%)	
>70 years	71 (44.4%)	10 (26.3%)	
Gender			
Male	82 (51.2%)	16 (42.1%)	0.311
Female	78 (48.8%)	22 (57.9%)	
BMI level			
Underweight (<18.5 kg/m^2^)	08 (05.0%)	07 (18.4%)	0.031 **
Normal (18.5 – 24.9 kg/m^2^)	59 (36.9%)	13 (34.2%)	
Overweight (25 – 29.9 kg/m^2^)	45 (28.1%)	11 (28.9%)	
Obese (≥30 kg/m^2^)	48 (30.0%)	07 (18.4%)	
Causes of CKD			
Not DM	31 (19.4%)	15 (39.5%)	0.008 **
DM	129 (80.6%)	23 (60.5%)	
On antihypertensive therapy			
No	41 (25.6%)	16 (42.1%)	0.044 **
Yes	119 (74.4%)	22 (57.9%)	
History of stroke			
No	120 (75.0%)	28 (73.7%)	0.867
Yes	40 (25.0%)	10 (26.3%)	
History of MI			
No	126 (78.8%)	33 (86.8%)	0.260
Yes	34 (21.2%)	05 (13.2%)	
History of cardiovascular disease			
No	84 (52.5%)	25 (65.8%)	0.139
Yes	76 (47.5%)	13 (34.2%)	
History of heart failure			
No	99 (61.9%)	26 (68.4%)	0.452
Yes	61 (38.1%)	12 (31.6%)	
HCV positive			
No	154 (96.2%)	30 (78.9%)	<0.001 **
Yes	06 (03.8%)	08 (21.1%)	
HBV positive			
No	147 (91.9%)	37 (97.4%)	0.235
Yes	13 (08.1%)	01 (02.6%)	
Type of dialysis therapy			
HD	121 (76.1%)	27 (71.1%)	0.518
HDF	38 (23.9%)	11 (28.9%)	
Type of vascular access			
AVF	58 (36.2%)	21 (55.3%)	0.085
AVG	03 (01.9%)	01 (02.6%)	
PC	99 (61.9%)	16 (42.1%)	

Ejection fraction, diastolic dysfunction, normal right and left atrial function, hypokinesia, pseudo-normalization, and hospitalization at 12 months were not statistically associated with CRP levels (all p>0.05) (Table 4).

**Table 4:** Relationship between the level of CRP and the baseline characteristics of the patients at King Abdulaziz Medical City and the South and North Dialysis Centers of King Abdullah Dialysis Program (cont’d.) (n=198) CRP: C-reactive protein

Factor	High N (%) ^(n=160)^	Normal N (%) ^(n=38)^	p-value
Ejection fraction (%)			
<25%	03 (02.1%)	01 (02.9%)	0.426
25 – 35%	08 (05.6%)	02 (05.9%)	
36 – 45%	15 (10.6%)	04 (11.8%)	
46 – 55%	94 (66.2%)	26 (76.5%)	
>55%	22 (15.5%)	01 (02.9%)	
Diastolic dysfunction			
No	82 (51.2%)	19 (50.0%)	0.890
Yes	78 (48.8%)	19 (50.0%)	
Right atrial normal			
No	33 (20.6%)	10 (26.3%)	0.444
Yes	127 (79.4%)	28 (73.7%)	
Left atrial normal			
No	96 (60.0%)	25 (65.8%)	0.510
Yes	64 (40.0%)	13 (34.2%)	
Hypokinesia present			
No	125 (78.1%)	24 (63.2%)	0.055
Yes	35 (21.9%)	14 (36.8%)	
Pseudo-normalization present			
No	129 (80.6%)	33 (86.8%)	0.372
Yes	31 (19.4%)	05 (13.2%)	
Hospitalization in 12 months			
No	60 (37.5%)	20 (52.6%)	0.087
Yes	100 (62.5%)	18 (47.4%)	

In the multivariate regression model, HCV positivity was the only independent significant factor associated with a low CRP level, whereas HCV-negative patients were five times more likely to exhibit high CRP levels (adjusted odds ratio 5.545; 95% confidence interval (CI) 1.637-18.789; p=0.006). In contrast, BMI, causes of CKD, antihypertensive therapy, and Kt/V had no significant effect after adjusting the regression model (Table 5).

**Table 5:** Multivariate regression analysis to determine the independent significant factor associated with high risk of CRP (n=198) DM: Diabetes mellitus; CRP: C-reactive protein

Factor	AOR	95% CI	p-value
BMI level			
Underweight (<18.5 kg/m^2^)	Ref		
Normal (18.5 – 24.9 kg/m^2^)	2.228	0.453 – 10.965	0.324
Overweight (25 – 29.9 kg/m^2^)	0.984	0.328 – 2.950	0.977
Obese (≥30 kg/m^2^)	1.323	0.442 – 3.961	0.617
Causes of CKD			
Not DM	Ref		
DM	0.765	0.306 – 1.912	0.567
On antihypertensive therapy			
No	Ref		
Yes	0.514	0.228 – 1.160	0.109
HCV negative			
Yes	5.545	1.637 – 18.789	0.006 **
No	Ref		
Kt/V	3.583	0.838 – 15.326	0.085

**Figure 1:**
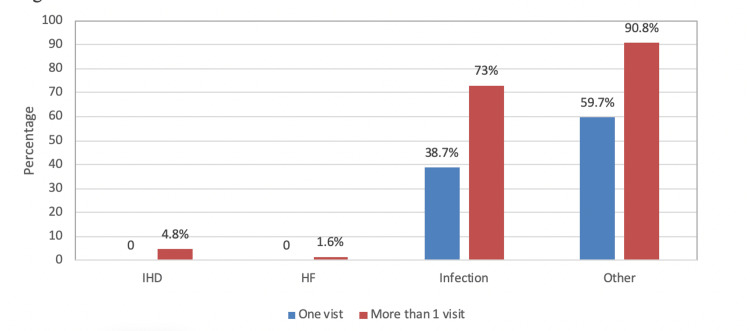
Frequency of hospitalization among the patients

Figure 1 illustrates the frequency of hospitalization among the patients, with infection being the most common cause.

## Discussion

Results of the present study demonstrated that the relationship between the CRP level and BMI, age, CCI, and DM was similar to those described in other studies that reported a positive correlation, with the exception of age and CCI, which did not reach a statistically significant level.

Although it is well established that CRP levels are associated with hypertension, a negative correlation between antihypertensive therapy and CRP levels was found, in contrast to other studies [13-16]. One hypothesis to explain these discrepancies is possible confounders, such as smoking, obesity, and compliance, which may contribute to high CRP levels despite antihypertensive drug therapy [17].

In relation to Kt/V, with higher values indicating the efficacy of the dialysis session, results of this study revealed that the link between high CRP levels and lower Kt/V values was statistically significant, and lower values of the latter have been associated with high morbidity and mortality rates, according to previous studies [18,19].

This study found that patients with a history of hospitalization in the previous 12 months exhibited higher CRP levels, with infections being the most common cause, and CRP levels were higher in those with PC access than in other types of access, consistent with several studies. Many studies have suggested that PC use increases the risk of hospital admission(s) and infections, which may explain why infections are the most common cause of hospitalizations [20-23].

Our findings revealed a negative correlation between HCV-positivity and CRP levels, with a statistically significant p-value in both chi-squared tests and multivariate regression analysis, similar to previous reports. This may be explained by the fact that HCV viral load diminishes the production of CRP by damaging hepatocytes [24,25].

Disrupted mineral metabolism is common in patients with CKD and is associated with high CRP levels and potential cardiovascular morbidity and mortality. In this analysis, we found no association between high CRP levels and disrupted mineral metabolism. In contrast to other studies, the mean values of calcium, phosphorus, PTH, and calcium/phosphorus products were all within the normal ranges, which may explain discrepancies in the findings. This suggests that adherence to the Kidney Disease Outcomes Quality Initiative (KDOQI) guideline varies and the probable use of phosphorus binders may have played a role in these findings [1,26,27].

Although not statistically significant, there was an association between high CRP levels and high ERI, supporting other studies that confirmed that high ERI was associated with cardiovascular mortality [28,29].

The presence of both pseudo-normalization and hypokinesis, which indicate diastolic and systolic dysfunction, respectively, was associated with high CRP levels in the present study, which can predict cardiovascular complications, including congestive heart failure according to a previous cohort study [30].

The primary limitation of this study was its cross-sectional design, which precludes any conclusions regarding causation. As such, further studies are needed to determine whether CRP levels should be routinely measured in patients undergoing HD.

## Conclusions

There was an association between CRP levels and BMI, DM, use of antihypertensive medications, and negative or undetectable HCV test results, with the latter being the only significant independent factor. These data suggest that patients with these characteristics are in an inflammatory state and are more prone to develop complications. Therefore, the implementation of CRP testing in clinical settings may be useful for the early detection of comorbidities in this patient population.
